# Nanomedicine‐boosting icaritin-based immunotherapy of advanced hepatocellular carcinoma

**DOI:** 10.1186/s40779-022-00433-9

**Published:** 2022-12-12

**Authors:** Yi Lu, Yue Gao, Huan Yang, Yong Hu, Xin Li

**Affiliations:** 1 Department of Polymeric Materials, School of Materials Science and Engineering, Tongji University, Shanghai, 201804 China; 2 Institute of Biotechnology, RWTH Aachen University, 52074 Aachen, Germany; 3 DWI-Leibniz-Institute for Interactive Materials e.V., 52056 Aachen, Germany; 4 College of Chemistry, Chemical Engineering and Biotechnology, Donghua University, Shanghai, 201620 China; 5 School of Pharmacy, Jiangsu University, Zhenjiang, 212013 Jiangsu China; 6 Institute of Technical and Macromolecular Chemistry, RWTH Aachen University, 52074 Aachen, Germany

**Keywords:** Icaritin, Nanomedicine, Advanced hepatocellular carcinoma, Immunotherapy, Clinical translation

## Abstract

Traditional treatments for advanced hepatocellular carcinoma (HCC), such as surgical resection, transplantation, radiofrequency ablation, and chemotherapy are unsatisfactory, and therefore the exploration of powerful therapeutic strategies is urgently needed. Immunotherapy has emerged as a promising strategy for advanced HCC treatment due to its minimal side effects and long-lasting therapeutic memory effects. Recent studies have demonstrated that icaritin could serve as an immunomodulator for effective immunotherapy of advanced HCC. Encouragingly, in 2022, icaritin soft capsules were approved by the National Medical Products Administration (NMPA) of China for the immunotherapy of advanced HCC. However, the therapeutic efficacy of icaritin in clinical practice is impaired by its poor bioavailability and unfavorable in vivo delivery efficiency. Recently, functionalized drug delivery systems including stimuli-responsive nanocarriers, cell membrane-coated nanocarriers, and living cell-nanocarrier systems have been designed to overcome the shortcomings of drugs, including the low bioavailability and limited delivery efficiency as well as side effects. Taken together, the development of icaritin-based nanomedicines is expected to further improve the immunotherapy of advanced HCC. Herein, we compared the different preparation methods for icaritin, interpreted the HCC immune microenvironment and the mechanisms underlying icaritin for treatment of advanced HCC, and discussed both the design of icaritin-based nanomedicines with high icaritin loading and the latest progress in icaritin-based nanomedicines for advanced HCC immunotherapy. Finally, the prospects to promote further clinical translation of icaritin-based nanomedicines for the immunotherapy of advanced HCC were proposed.

## Background

Hepatocellular carcinoma (HCC) is the third leading cause of cancer-related deaths worldwide. The 5-year survival rate of HCC patients is 12%, which is much lower than that of other cancers [1]. Conventional therapies including surgical resection, transplantation and radiofrequency ablation, have been widely adopted for the treatment of early-stage HCC. However, 80% of HCC patients are diagnosed at an advanced stage due to the lack of specific symptoms [2, 3]. Advanced HCC has a complex microenvironment consisting of cancer, stromal and immune cells, as well as extracellular matrix (ECM). The interplay between these components within the HCC microenvironment leads to fibrosis, angiogenesis, and inflammation in the liver, driving the progression and metastasis of advanced HCC [4]. Most advanced HCCs are difficult to be completely resected, and therefore systemic chemotherapy is the clinically preferred therapeutic approach. Several drugs, such as sorafenib, regorafenib, and lenvatinib, have been approved in clinical practice. Yet, chemotherapy offers only a modest overall survival rate due to the serious side effects of long-term medication [5, 6]. Moreover, advanced HCC patients suffering from liver functions impairments are often more vulnerable to drug-associated toxicity. In addition, advanced HCC increases the expression of proteins that can generate drugs chemoresistance [7]. Hence, there is an urgent need to develop novel drug delivery and therapeutic approaches according to the properties of advanced HCC.

In the recent decade, immunotherapy has emerged as a powerful method against many types of cancers due to its minimal side effects and long-lasting therapeutic memory effects [8]. Along with acceleration in clinical approvals, several modalities of cancer immunotherapy have achieved significant progress [9]. Recently, scientists explored the feasibility of immunotherapy for advanced HCC, such as immune checkpoint blockade (ICB) [e.g., the monoclonal antibodies (mAbs) tremelimumab and nivolumab], which have enhanced survival rates in advanced HCC patients [10, 11]. However, chronic inflammation and antigenic stimulation during the progression of advanced HCC lead to an immunosuppressive microenvironment with the functionally impaired effector T cells, as well as a high level of infiltration and large accumulation of suppressive myeloid cells [12, 13]. Previous research reported that ICB treatment for advanced HCC using a programmed death-ligand 1 (PD-L1) antibody had a low remission rate of less than 20% [14]. It is important to reduce the recruitment of immunosuppressive cells in the HCC microenvironment. Thus, overcoming the immunosuppression of advanced HCC with newly-explored immunomodulators would be one of the key ways to improve the therapeutic outcomes.

**Fig. 1:**
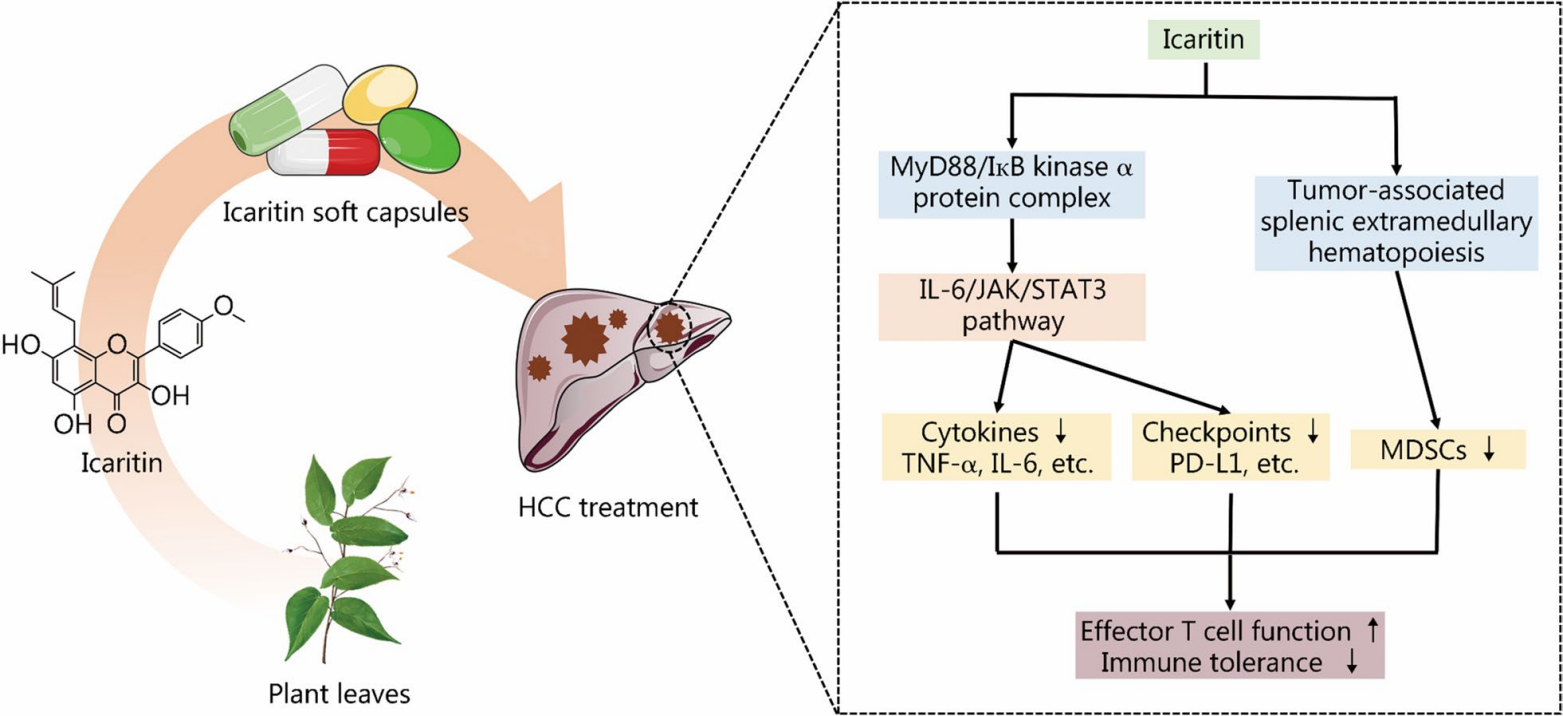
Icaritin soft capsule for immunotherapy of advanced HCC. Icaritin can be extracted from Epimedii Folium or produced by hydrolyzing the other major flavonoids in Epimedii Folium. The icaritin soft capsule formulations can be applied for immunotherapy of advanced HCC by interacting with the MyD88/IкB kinase α protein complex, suppressing IL-6/JAK/STAT3 signaling pathway, and reducing the generation of cytokines (e.g., TNF-α and IL-6) and the expression of immune checkpoints (e.g., PD-L1). Moreover, icaritin can inhibit the bioactivity of MDSCs by down-regulating the tumor-associated splenic extramedullary hematopoiesis. Finally, effector T cell function is enhanced and the immune tolerance in advanced HCC is alleviated, which ultimately improve the efficacy of icaritin-based immunotherapy in advanced HCC. HCC hepatocellular carcinoma, MyD88 myeloid differentiation factor 88, IL-6 interleukin-6, JAK Janus kinase, STAT3 signal transducer and activator of transcription 3, TNF-α tumor necrosis factor-α, PD-L1 programmed death-ligand 1, MDSCs myeloid-derived suppressor cells

The prenylated flavonoid icaritin, a traditional Chinese medicine, is an active natural compound derived from Epimedii Folium (Yinyanghuo in Chinese) (Fig. 1). Icaritin has been reported to display enormous potential to treat various diseases. In particular, icaritin and its derivatives play an anticancer role by triggering cell apoptosis, modulating the cell cycle and hormone signaling, inhibiting cancer angiogenesis and metastasis, suppressing the growth of cancer stem cells, and immunomodulation [15]. In the last decade, it was found that icaritin displays the excellent therapeutic efficacy on advanced HCC [16, 17]. Notably, recent study indicated that icaritin represents a potential immunomodulator with favorable biosafety, prolonged survival rate in advanced HCC patients [18]. In January 2022, icaritin soft capsules were approved as an immunomodulatory agent by the NMPA of China for the treatment of advanced HCC based on the positive results from clinical phase III trials (NCT03236636, NCT03236649) [18, 19].

Despite considerable progress, the immunotherapeutic effect of icaritin in vivo for advanced HCC is still impaired by its low bioavailability and delivery efficiency. Pharmacokinetic studies have shown that only 4.33% of icaritin is rapidly absorbed into the blood of rats, and the overall bioavailability was as low as 2% due to its limited aqueous solubility and low permeability [20, 21]. In recent years, the development of icaritin-based nanomedicines has provided a solution for enhancing therapeutic efficacy with low side effects. Various nanomedicines have been developed to improve the bioavailability, prolong blood circulation, augment targeted accumulation, and elevate tumor penetration of icaritin. In addition, stimuli-responsive nanocarriers allow the tailored release of icaritin in advanced HCC with excellent spatial, temporal and dosage control. Therefore, these functionalized nanomedicines exhibit favorable biodistribution of icaritin and reduced toxicity to healthy tissues [22–25]. Moreover, these nanomedicines may help to raise the antigenicity of advanced HCC for a sustained cancer-immunity cycle. Remarkably, several polymeric nanocarriers including polymer conjugates, liposomes and micelles have been approved for clinical practice for the treatment of cancers worldwide. The combination of the clinically approved nanocarriers with icaritin would be one of the most promising ways to create the icaritin-based nanomedicines with clinical translation potential for the treatment of advanced HCC.

In this perspective, we summarize the latest progress of icaritin and icaritin-based nanomedicines for enhanced immunotherapy of advanced HCC. First, several icaritin preparation methods are compared. Second, the HCC immune microenvironment and the mechanisms of icaritin against advanced HCC are interpreted. Third, the design of icaritin-based nanomedicines with high icaritin loading efficiency is described. Fourth, icaritin-based nanomedicines for improved immunotherapy of advanced HCC are discussed. Finally, the prospects for facilitating the further clinical translation of icaritin-based nanomedicines are proposed.

## Preparation of active icaritin

Icaritin and some major flavonoids (e.g., icariin, epimedin A, epimedin B, epimedin C, and baohuoside I) in Epimedii Folium share the same fundamental skeleton but have different glycosyl substitutions at the C-3 and C-7 positions [26]. The content of icaritin, which is an aglycone of the epimedium flavonoids without any sugar moieties, is lower than 0.1%, while icariin is the most abundant flavonoid in Epimedii Folium [27, 28].

For the production of icaritin, several methods including column chromatography, chemical synthesis, and acid and enzymatic hydrolysis have been developed. However, column chromatography, the commonly used method for icariin preparation, is not feasible for large-scale production due to the low amount of icaritin found in natural plants [29]. The industrial application of chemical synthesis remains difficult due to the tedious procedures (more than 8 steps), harsh reaction conditions (high temperature of 110 ℃), low yield (less than 23%), and adverse effects on product activity [30, 31]. In addition, acid hydrolysis of icariin tends to generate various byproducts, such as baohuoside I, icariside I, maohuoside A, and anhydroicaritin 3-O-rhamnoside [32]. Recently, enzymatic hydrolysis has been utilized to obtain icaritin, as it has the advantages of remarkable selectivity, mild reaction conditions, high efficiency and environmental friendliness. In enzymatic reactions, icaritin is produced by removing the sugar moieties at both the C-3 and C-7 positions of other flavonoids, such as icariin. For instance, icariin has been hydrolyzed into icaritin with the high productivity of 86.2% and a corresponding molar conversion rate of 91.2% using GH78 α-L-rhamnosidase and recombinant β-glucosidase through a two-stage transformation [33].

Although enzymatic catalysis is superior to other preparation methods, the productivity of icaritin decreases when enzyme activity and stability are impaired after a long reaction time. To further improve enzymatic catalytic efficiency, a directed evolution campaign can be used to generate enzyme variants that tolerate high reaction temperatures, product inhibition, and organic solvents. This strategy has been previously verified to improve the thermostability, activity, and glucose tolerance of β-glucosidase and α-L-rhamnosidase [34, 35]. Likewise, enzyme immobilization technology is another option to improve the stability and reusability of enzymes [36, 37]. Dong et al. [36] reported that two thermostable glycosidases (β-glucosidase DthBgl3 and α-L-rhamnosidase DthRha) were successfully immobilized on 1000NH amino resin. The immobilized DthBgl3 and DthRha transformed the total flavonoids extract from epimedium completely into icaritin with a molar conversion rate of 87.21% and productivity of 141 mg/(L·h) after 15 cycles of repeated use. Moreover, noninvasive green solvents, such as poly ethylene glycol (PEG), polyacrylate, ionic liquids, and deep eutectic solvents, have been successfully applied to improve the enzymatic hydrolysis of other flavonoids, such as rutin, and can be employed to dissolve poorly water-soluble icariin and maintain enzyme activity by virtue of their biocompatibility and biosecurity [38, 39]. The combination of the aforementioned strategies, such as conducting deglycosylation in a hydrolysis system containing immobilized β-glucosidase and α-L-rhamnosidase variants with high performance and deep eutectic solvent-dissolved icariin, would significantly improve hydrolysis efficiency and thus increase the productivity of icaritin.

Additionally, biosynthetic methods with engineered microbial strains offer alternative ways to generate icaritin from glucose, which could facilitate the industrial-scale production of icaritin and other prenylflavonoids [40]. The productivity of icaritin can be further increased by engineering microbial strains and optimizing the fermentation conditions.

## HCC immune microenvironment

During HCC progression, HCC cells reprogram their metabolism and interact with stromal cells and the complex ECM to shape an immunosuppressive microenvironment for immune privilege. First, compared to tumors with high tumor mutation burden (TMB), such as melanoma, HCC shows a relatively low TMB [41]. Correspondingly, the antigenicity of HCC is generally unfavorable for a sustained cancer-immunity cycle that requires enough tumor neoantigen to stimulate antigen presenting cells (APCs) and T cells. Additionally, local liver cells are actively involved in tumor immune tolerance. For example, Kupffer cells in the liver produce the inhibitory cytokine interleukin-10 (IL-10) and indoleamine 2,3-dioxygenase (IDO) to activate immunosuppressive regulatory T cells (Tregs), and hepatic stellate cells (HSCs) and liver sinusoidal endothelial cells (LSECs) also drive the accumulation of myeloid-derived suppressor cells (MDSCs) and Tregs [42]. The densities of these immunosuppressive types of immune cells within the immune microenvironment correlate with the poor prognosis of advanced HCC. For example, the increased infiltration of Tregs in the liver is associated with the dysfunction of T cell-mediated tumor surveillance. MDSCs support the progression of HCC by promoting the production of vascular endothelial growth factor (VEGF), which further facilitates the vascularization and angiogenesis of tumors. As a result, multiple immunosuppressive factors are constantly observed in the immune microenvironment. Tumor infiltrating lymphocytes (TILs) in the advanced HCC immune microenvironment tend to be dysfunctional with the enhanced expression of co-inhibitory molecules, including programmed death-1 (PD-1), cytotoxic T lymphocyte associated antigen 4 (CTLA-4), lymphocyte-activation gene 3 (LAG-3), and T cell immunoglobulin and mucin domain containing-3 (TIM-3) [43]. Additionally, a decreased CD8^+^/CD3^+^ T cell ratio and CD56^+^ natural killer (NK)/natural killer T (NKT) cell infiltration can be found in the HCC differentiation.

## Anti-HCC mechanisms of icaritin

Icaritin exerts therapeutic efficacy on advanced HCC through both chemotherapy and immunomodulation, and acts both on cancer cells and immune cells, especially MDSCs. As a chemotherapeutic compound, icaritin can induce cell apoptosis via a caspase-dependent pathway [44]. In recent work, icaritin was found to promote apoptosis of HCC cells by down-regulating alpha-fetoprotein gene expression [35]. In addition, Wang et al. [17] found that icaritin could trigger cellular senescence by inducing the production of reactive oxygen species (ROS) and DNA damage. Importantly, a lower amount of icaritin was needed to trigger cellular senescence than to induce cell death, which can avoid the severe side effects of drugs. Likewise, icaritin was demonstrated to potentiate doxorubicin (DOX)-induced immunogenic cell death (ICD) in advanced HCC and thus improve the immune response by inducing mitophagy and apoptosis [45, 46].

Recently, a growing amount of evidence has suggested that icaritin can be applied for advanced HCC immunotherapy by modulating the immune system. As shown in Fig. 1, the immunomodulatory activities of icaritin are mainly associated with the interleukin-6 (IL-6)/Janus kinase (JAK)/signal transducer and activator of transcription 3 (STAT3) signaling pathway. Icaritin interacts with the myeloid differentiation factor 88 (MyD88)/IкB kinase α protein complex, which further suppresses the downstream IL-6/JAK/STAT3 signaling pathways and influences the secretion of cytokines and the expression of immune checkpoint molecules as well as the differentiation of immune cells. Specifically, the involvement of icaritin in advanced HCC treatment can relieve immunosuppression by reducing the generation of the inflammatory cytokines tumor necrosis factor-α (TNF-α) and IL-6, down-regulating the expression of the PD-L1 checkpoint in MDSCs and neutrophils, and restoring the function of interferon-γ (IFN-γ)^+^ CD8^+^ T cells [2, 47, 48]. In addition, icaritin can directly downregulate the tumor-associated splenic extramedullary hematopoiesis (EMH), and thereby reduce the generation, activation and accumulation of MDSCs in tumor sites as well as recover the functions of effector T cells. This can then coordinate with PD-1 antibody-based ICB for enhanced antitumor responses in mouse tumor models [49]. Encouragingly, in a clinical phase I trial, icaritin was found to improve the survival rate by inducing the changes in immune biomarkers and immunosuppressive myeloid cells in patients with advanced HCC [2]. These findings not only help to speed up the approval of icaritin as an immunomodulator for advanced HCC, but also provoke thoughts for the development of other anti-IL-6/JAK/STAT3 drugs for cancers. Despite the abovementioned progress, the underlying mechanism of icaritin in advanced HCC treatment is still elusive.

## Design of icaritin-based nanomedicines

Nanocarriers have been considered to enhance the immunotherapy of icaritin for advanced HCC. The design of such nanocarriers is closely related to the physicochemical properties of icaritin. Icaritin possesses hydrophobic properties and contains active groups, such as phenolic hydroxyl moieties and benzene rings, which can form covalent and/or noncovalent interactions with nanocarriers. In addition, icaritin exhibits a negative charge under a physiological environment [50]. To achieve high loading efficiency, several kinds of suitable nanocarriers have been designed.

Micelles or liposomes with hydrophobic regions have been utilized to load icaritin through hydrophobic interactions. For instance, icaritin-loaded micelles were produced by encapsulating icaritin in the hydrophobic core of poly lactic-*co*-glycolic acid (PLGA) [45]. Additionally, nanocarriers with hydrogen bond acceptors or donors and aromatic planes can form hydrogen bonds and π-π interactions with icaritin [51, 52]. For example, the hydrogen bonding interaction between icaritin and a polysaccharide carrier was adopted to form icaritin-loaded pectin micelles [52]. Several cationic nanocarriers can also encapsulate icaritin through electrostatic interactions [53, 54]. In addition, icaritin can also be covalently conjugated with nanocarriers. A functionalized hyaluronic acid/collagen hydrogel was prepared by the formation of a disulfide bond between thiolated icariin and hyaluronic acid, which can be cleaved by the reductant glutathione that is secreted from cells [55]. Moreover, icaritin-based nanomedicines can be designed to release icaritin under certain conditions. Notably, a near infrared (NIR) light-responsive nanocarrier was designed for the controlled release of icariin [56]. In this study, icariin was loaded into mesoporous silica, and the formed nanomedicines were further covered with β-cyclodextrin (β-CD) through the linker 4-(hydroxymethyl)-3-nitrobenzoic acid (ONA). Upon 980 nm NIR light irradiation, ONA was photocleaved and the removal of β-CD was triggered, resulting in the release of icariin.

## Icaritin-based nanomedicines for improved immunotherapy of advanced HCC

Although icaritin soft capsules have been approved for advanced HCC treatment via oral administration, there are some limitations that have arisen from the properties of icaritin and administration route. First, oral administration imposes high demands on the solubility and permeability of drugs, and is thus not suitable for icaritin. In addition, the oral administration of icaritin faces some issues, including instability in the luminal fluid, insolubility in the intestinal tract, poor absorption in the mucous and cell membrane, and loss of bioactivity after first pass drug elimination process [57]. Taken together, all these factors contribute to the low oral bioavailability of icaritin (2%) [21]. Consequently, compared with other anti-HCC agents [250 mg/(person‧d) for gefitinib and 800 mg/(person‧d) for sorafenib], the larger dose of icaritin required [1200 mg/(person‧d)] may lead to severe side effects. Intravenous injection bypasses absorption barriers and avoids first pass drug elimination process. Moreover, intravenous injection is suitable for the drugs that are poorly absorbed by the gastrointestinal tract. Intravenous injection can be applied to overcome the problems from oral administration and thus obtain satisfactory therapeutic efficacy with a lower dose. A representative study on the intravenous injection of the anti-HCC agent anlotinib was performed by Luo et al. [58], which showed that a greatly reduced dose (1/10 of the oral dose) produced a significantly improved anti-HCC effect.

**Fig. 2:**
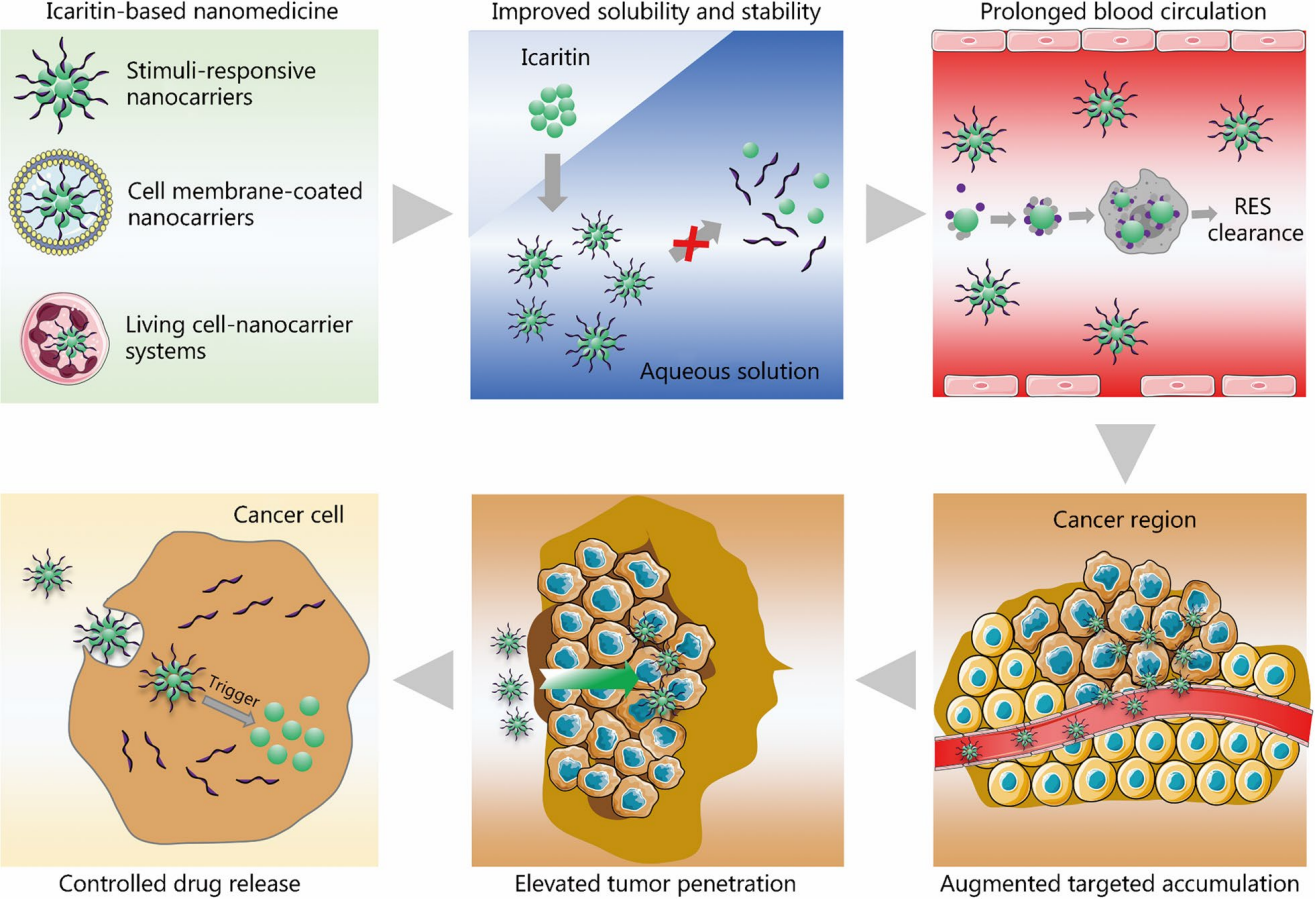
Icaritin-based nanomedicines for improved immunotherapy of advanced HCC. Smart drug delivery systems including stimuli-responsive nanocarriers, cell membrane-coated nanocarriers, and living cell-nanocarrier systems, can be designed to load icaritin. The formed icaritin-based nanomedicines show improved bioavailability and delivery efficiency as well as alleviated immunogenicity. Icaritin-based nanomedicines could enhance the immunotherapy of advanced HCC by improving drug solubility and stability, prolonging blood circulation, augmenting targeted accumulation, elevating tumor penetration and controlling drug release. Part of this figure was created partially utilizing the templates on https://smart.servier.com/ as a reference. HCC hepatocellular carcinoma, RES reticuloendothelial system

Recent studies have shown that nanocarriers have emerged as a novel toolbox for further improving the bioavailability and delivery efficiency of drugs as well as alleviating their toxicity of drugs during intravenous injection [59–62]. A variety of functionalized nanocarriers have been designed to improve drug solubility and stability, prolong blood circulation, augment targeted accumulation, elevate tumor penetration, and control drug release (Fig. 2). For example, PEG, peptides, zwitterions, and various ligands can be used to modify nanocarriers to render nanomedicines with desired functionality to avoid the clearance by the reticuloendothelial system (RES), and to cross the biological barriers in the body. However, the capabilities to cross multiple biological barriers simultaneously might be achieved by further endowing functionalized nanocarriers with size- or charge-reversible properties [63, 64]. For advanced HCC, several nanomedicines containing DOX [2], paclitaxel [65], simvastatin [66], sorafenib [67] and arsenic trioxide [68] have been developed to improve the chemotherapeutic effects. Recently, nanomedicines loaded with siRNA [69] and CRISPR/Cas system [70] were fabricated to increase the chemotherapeutic sensitivity in advanced HCC. Moreover, several drugs have been combined into a single nanoplatform to further improve the efficacy and reduce the toxicity of drugs during the treatment of advanced HCC [71].

**Fig. 3:**
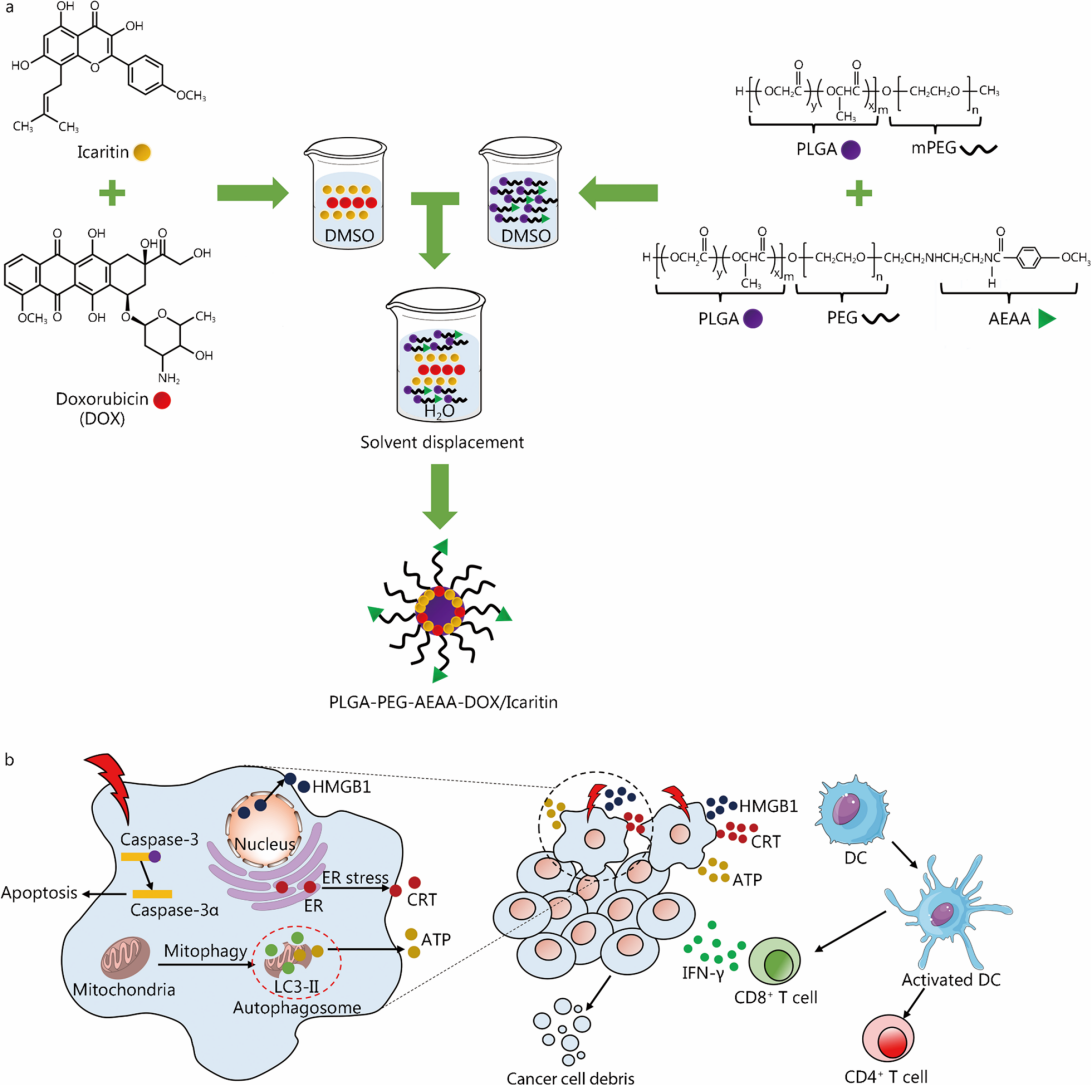
Icaritin-based nanomedicines for inducing ICD in advanced HCC. **a** PLGA-PEG-AEAA NPs were prepared by a solvent displacement technique to load icaritin and DOX. **b** The produced NPs displayed targeted delivery of icaritin and DOX, and efficiently improved the anti-HCC effect by remodeling the immunosuppressive tumor microenvironment and triggering a robust immune memory response. Reprinted with the permission from Ref. [45] Copyright © 2020, American Chemical Society. ICD immunogenic cell death, HCC hepatocellular carcinoma, PLGA poly lactic-co-glycolic acid, PEG poly ethylene glycol, mPEG monomethoxy poly ethylene glycol, AEAA aminoethyl anisamide, DOX doxorubicin, HMGB1 high mobility group box 1, ER estrogen receptor, CRT calreticulin, LC3-II microtubule-associated protein light chain 3 II, DC dendritic cell, IFN-γ interferon-γ

Nanomedicines are expected to solve several existing issues that icaritin faces during the treatment of advanced HCC, including low bioavailability, limited delivery efficiency, and a large required dose. Recent preclinical studies have proven that the applications of icaritin-based nanomedicines could boost the therapeutic efficacy against advanced HCC [45, 72]. The area under the curve (AUC_0–24_) and C_max_ of the icaritin-based nanomedicine were significantly higher than those of free icaritin, indicating that the bioavailability of the icaritin-based nanomedicines was greatly enhanced [21, 73]. In 2021, Guo et al. [72] reported that icaritin and coix seed oil co-loaded lipid complexes (IC-ML) displayed elevated penetration and growth inhibition of advanced HCC. Moreover, Yu et al. [45] developed (poly lactic-*co*-glycolic acid)-(poly ethylene glycol)-aminoethyl anisamide nanoparticles (PLGA-PEG-AEAA NPs) for highly efficient immunotherapy of advanced HCC by co-delivery of icaritin and DOX (Fig. 3). The designed nanomedicines extended the half-life times (t_1/2_) values of icaritin and DOX significantly from 0.41 and 0.14 h (free drugs) to 1.65 and 1.96 h, respectively. The AUC of the nanomedicines was three-fold higher than those of their free forms. These results indicated that the icaritin and DOX-loaded nanomedicine displayed improved blood circulation and bioavailability. In addition, the accumulation of PLGA-PEG-AEAA NPs in the tumor region was accelerated via enhanced permeability and retention (EPR) effect due to the suitable size of approximately 100 nm. At the same time, AEAA, which can bind the σ − 1 receptor expressed in tumor tissue, rendered the delivery system with the property to actively target the tumor region. Such active targeting together with the EPR effect reduced the off-target risks. Moreover, PLGA NPs can be degraded in the acidic tumor region, enabling controlled drug release. In animal experiments, augmented tumor growth inhibition and an increased survival rate were observed in tumor-bearing mice. Additionally, this work revealed the collaborative mechanism of icaritin with DOX for enhancing the antitumor immune response. Clearly, on one hand, icaritin induces ICD markers by promoting cell mitophagy, and on the other hand, it synergizes with the DOX-based ICD effects, together remodeling the immunosuppressive microenvironment of advanced HCC. Additionally, only a low-dose of each drugs was needed in this co-delivery strategy so that the vulnerable liver could be protected.

Despite the significant progress made, icaritin-based nanomedicines suffer from systematic immunogenicity leading to the inevitable clearance by RES and subsequently limited delivery efficiency and tumor accumulation. To address these limitations, the application of natural exosomes extracted from fetal bovine serum to delivery icariin was explored by Dong et al. [74]. The icariin-loaded exosomes displayed enhanced bioavailability and promoted osteoblast proliferation due to their low immunogenicity and reduced sensitivity to RES stress. In addition, cell membrane-coated nanomedicines combine the versatility of synthetic nanocarriers and intrinsic functionalities of natural cell membranes, contributing to RES clearance evasion and targeted delivery to specific sites. For example, a nanomedicines coated with both 4T1 tumor cell and dendritic cell membranes exhibited both blood circulation ability and immunotherapeutic effects [75]. This biomimetic complex can serve as a nanovaccine to effectively accumulate in both tumors and lymph nodes for intrinsic immune activation, eventually resulting in the inhibition of tumor growth, prevention of metastasis, and generation of immunological memory. In recent years, living cell-nanomedicine systems have been established for cancer treatment [76]. For instance, macrophage cell-nanomedicine systems with highly-efficient tumor homing abilities were utilized to deliver DOX-silica nanocomplex (DSN) [77]. Once the DSN was transported to tumors via chemotactic migration, DOX was released from DSN to kill cancer cells. These aforementioned novel biomimetic drug delivery strategies would be an ideal solution to further increase the tumor accumulation of icaritin and minimize toxicity to normal tissues.

In addition, the application of nanomedicines would benefit cancer immunotherapy through optimal design principles [78–80]. For example, composite carriers with photodynamic, chemodynamic, sonodynamic and other dynamic properties to trigger ICD could be further constructed to deliver icaritin to increase the antigenicity of HCC, maximize the immunoactivities of icaritin, and reduce the immunosuppression in advanced HCC [81, 82].

**Fig. 4:**
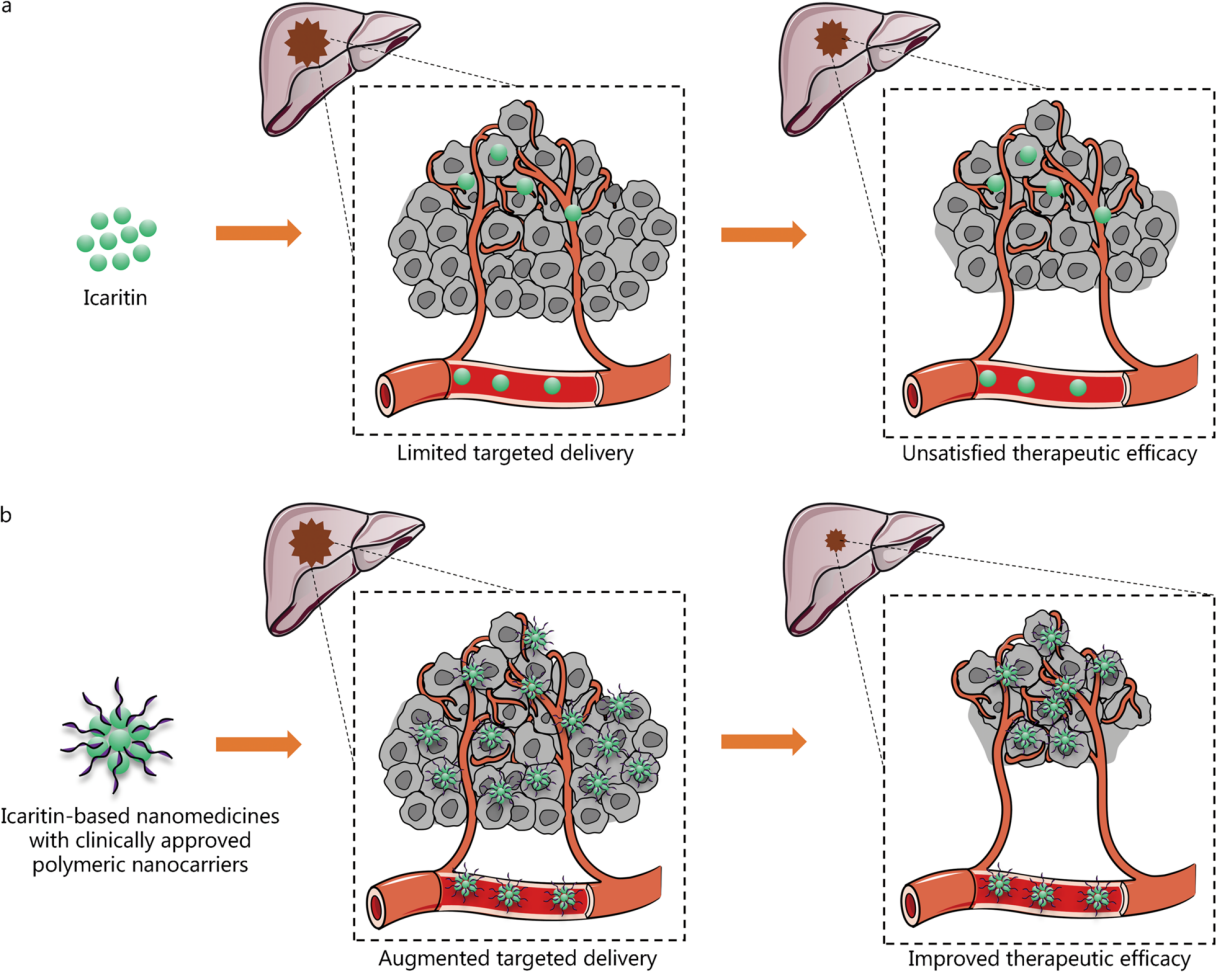
Immunotherapy of advanced HCC using icaritin (**a**) or icaritin-based nanomedicines (**b**). Clinically approved nanocarriers (e.g., polymeric conjugates, micelles and liposomes) can be used to deliver icaritin in clinical practice. Compared with free icaritin, icaritin-based nanomedicines display augmented targeted delivery in vivo, resulting in improved therapeutic efficacy for immunotherapy of advanced HCC. Part of this figure was created partially utilizing the templates on https://smart.servier.com/ as a reference. HCC hepatocellular carcinoma

With constant efforts and preliminary outcomes toward the development of functionalized nanomedicines, 14 systemically administered nanomedicines based on polymeric conjugates, micelles and liposomes have been approved for anti-cancer use in clinical practice worldwide [83]. This has inspired the utility of these nanocarriers to deliver icartitin for advanced HCC treatment (Fig. 4) [84]. Despite this, the interplay between the HCC microenvironment and nanomedicines should be taken into consideration in clinical practice. The nanomedicines used for the advanced HCC treatment should be carefully assessed in terms of their own burden on liver tissue, and the fate of icaritin as well as the nanomedicine within the complex HCC microenvironment should be studied, especially how they affect the infiltration of immunosuppressive cells and the expression of co-inhibitory molecules. Only when the clinical demands are considered can the success of icaritin-based nanomedicines for clinical translation in the treatment of advanced HCC be realized.

## Conclusions

In summary, icaritin-based nanomedicines display enormous potential for immunotherapy of advanced HCC. Herein, we first compared the preparation methods of icaritin, and enzymatic hydrolysis and biosynthesis represent two promising methods to produce icaritin on a large-scale with great potential in industrial applications. Then, the HCC immune microenvironment and the treatment mechanisms of icaritin for advanced HCC were interpreted. The immunomodulatory mechanism related to the IL-6/JAK/STAT3 signaling pathway in advanced HCC immunotherapy was highlighted. Additionally, the design of icaritin-based nanomedicines for high icaritin loading efficiency was discussed. The development of icaritin-based nanomedicines for improved immunotherapy of advanced HCC was also presented. Moreover, further directions for the clinical translation of icaritin-based nanomedicines in advanced HCC treatment were proposed.

With the development of functionalized nanocarriers, icaritin-based nanomedicines could, in principle, be constructed to improve therapeutic efficacy against advanced HCC. Several potential directions are put forward for challenges that exist in the clinical applications. First, a deep investigation should be carried out regarding the immunomodulatory mechanisms of icaritin for advanced HCC treatment as well as the crosstalk between cancer cell death pathways. Second, functionalized nanomedicines with photodynamic, chemodynamic, sonodynamic or other dynamic properties could be used to increase the antigenicity of HCC to synergize with the immune activities of icaritin. Third, the in vivo biocompatibility, delivery efficiency, and therapeutic effects of icaritin-based nanomedicines should be comprehensively investigated. Fourth, to relieve the nanotoxicity of nanomedicines, a feasible industrialization route and safety profile of icaritin-based nanomedicines should be explored. Fifth, clinical trials of icaritin-based nanomedicines should be preferentially performed with clinically approved nanocarriers. Sixth, prodrug nanosystems with the integrated advantages of both nanocarriers and prodrugs might be exploited for efficient immunotherapy with decreased side effects. Several prodrug nanomedicines are currently in clinical trials, such as the NK012 for the treatment of colorectal cancer (NCT00542958) [85]. Thus, it is anticipated that the construction of icaritin prodrug nanosystems could potentially enhance the immunotherapy of advanced HCC in clinical practice [86].

Last but not least, the complicated designs of nanomedicines do not necessarily mean more barriers in vivo can be overcome. There is a possibility that the complicated nanomedicines could have negative effects on icaritin therapeutic efficacy. Thus, additional efforts should be made to balance the functionality and complexity of nanomedicine design [87]. It is also urgent to better understand the interaction between the nanomedicine and the complicated physiological environment. In the case of advanced HCC, it is especially important to clarify the own burdens of nanomedicines on the vulnerable liver and their interplay in the HCC microenvironment. Along with desirable functionality and improved physicochemical properties, frameworks or databases for nanotoxicity and nano-bio interactions should be further established to serve as guidelines for the general applications of nanomedicines and the development of icaritin-based nanomedicines. We believed that, with rational design of icaritin-based nanomedicines considering the abovementioned aspects, the therapeutic effects for advanced HCC or other human diseases can be significantly improved and the barriers of icaritin in terms of both the systemic circulation in the body and on the way toward clinical translation can be overcome.

## Data Availability

Not applicable.

## Abbreviations

APC: Antigen presenting cell
AUC: Area under the curve
CTLA-4: Cytotoxic T lymphocyte associated antigen 4
DOX: Doxorubicin
DSN: DOX-silica nanocomplex
ECM: Extracellular matrix
EMH: Extramedullary hematopoiesis
EPR: Enhanced permeability and retention
HCC: Hepatocellular carcinoma
HSCs: Hepatic stellate cells
ICB: Immune checkpoint blockade
ICD: Immunogenic cell death
IC-ML: Icaritin and coix seed oil co-loaded lipid complexes
IDO: Indoleamine 2,3-dioxygenase
IFN-γ: Interferon-γ
IL-6: Interleukin-6
JAK: Janus kinase
LAG-3: Lymphocyte-activation gene 3
LSECs: Liver sinusoidal endothelial cells
mAb: Monoclonal antibody
MDSCs: Myeloid-derived suppressor cells
MyD88: Myeloid differentiation factor 88
NIR: Near infrared
NK: Natural killer
NKT: Natural killer T
NMPA: National medical products administration
ONA: 4-(hydroxymethyl)-3-nitrobenzoic acid
PD-1: Programmed death-1
PD-L1: Programmed death-ligand 1
PEG: Poly ethylene glycol
PLGA: Poly lactic-*co*-glycolic acid
PLGA-PEG-AEAA NPs: (poly lactic-*co*-glycolic acid)-(poly ethylene glycol)-aminoethyl anisamide nanoparticles
RES: Reticuloendothelial system
ROS: Reactive oxygen species
STAT3: Signal transducer and activator of transcription 3
TILs: Tumor infiltrating lymphocytes
TIM-3: T cell immunoglobulin and mucin domain containing-3
TMB: Tumor mutation burden
TNF-α: Tumor necrosis factor-α
Tregs: Regulatory T cells
VEGF: Vascular endothelial growth factor
β-CD: β-cyclodextrin
