# Correction to: Nanomedicine‐boosting icaritin-based immunotherapy of advanced hepatocellular carcinoma

**DOI:** 10.1186/s40779-023-00440-4

**Published:** 2023-01-25

**Authors:** Yi Lu, Yue Gao, Huan Yang, Yong Hu, Xin Li

**Affiliations:** 1 Department of Polymeric Materials, School of Materials Science and Engineering, Tongji University, Shanghai, 201804 China; 2 College of Chemistry, Chemical Engineering and Biotechnology, Donghua University, Shanghai, 201620 China; 3 School of Pharmacy, Jiangsu University, Zhenjiang, 212013 Jiangsu China

**Correction to: Military Medical Research (2022) 9:69** 10.1186/s40779-022-00433-9

Following publication of the original article [1], it was found there are some errors in the article.

The affiliations of the authors are incorrect, the correct author list with correct affiliations is shown below.

The Funding information should be updated to the below one:

This work was supported by the National Natural Science Foundation of China (52103181, 81873196), and the Fundamental Research Funds for the Central Universities (22120220075).

The original paper is updated.
